# Practice of switch from intravenous to oral antibiotics

**DOI:** 10.1186/2193-1801-3-717

**Published:** 2014-12-09

**Authors:** Zeina M Shrayteh, Mohamad K Rahal, Diana N Malaeb

**Affiliations:** School of Pharmacy, Department of Clinical Pharmacy, Lebanese International University, Mazraa, 146404 Beirut Lebanon; School of Pharmacy, Department of Pharmaceutical Sciences, Lebanese International University, Mazraa, 146404 Beirut, Lebanon

**Keywords:** Intravenous to oral antibiotics, Switch therapy, IV to PO conversion, Clinical outcomes, Antibiotic stewardship

## Abstract

Hospitalized patients initially on intravenous antibiotics can be safely switched to an oral equivalent within the third day of admission once clinical stability is established. This conversion has many advantages as fewer complications, less healthcare costs and earlier hospital discharge. The three types of intravenous to oral conversion include sequential, switch, and step-down therapy. The aim of the study was to evaluate the practice of switching from intravenous to oral antibiotics, its types and its impact on the clinical outcomes. This was a retrospective observational study conducted in three Lebanese hospitals over a period of six months. Adult inpatients on intravenous antibiotics for 2 days and more were eligible for study enrollment. Excluded were patients admitted to care or surgery units, or those with gastrointestinal diseases, infections that require prolonged course of parenteral therapy, or malignancies.

The study showed that among 452 intravenous antibiotic courses from 356 patients who were eligible for conversion, only one third were switched and the others continued on intravenous antibiotics beyond day 3 (P <0.0001). The mean duration of intravenous therapy of converted patients was markedly shorter than the non-converted (P <0.0001) with no significant change in the mean length of stay. Fluoroquinolones and macrolides were the most commonly converted antibiotics. However, the sequential therapy was the major type of conversion practiced in this study. Based on the study findings, a significant proportion of patients can be considered for switch. This emphasizes an important gap in the field of conversion from intravenous to oral antibiotic therapy and the need for integration and reinforcement of the appropriate Antibiotic Stewardship Programs in hospitals.

**Electronic supplementary material:**

The online version of this article (doi:10.1186/2193-1801-3-717) contains supplementary material, which is available to authorized users.

## Introduction

The appropriate use of antibiotics depends on the selection of an agent capable of achieving a desired serum concentration to target the presumed organism at the site of infection with an acceptable safety profile (Burke 2003; Davey et al. 2006). Inappropriate and unnecessary antimicrobial usage leads to an increase in healthcare costs and pathogen resistance (Spellberg et al. 2008).

Antimicrobial Stewardship Programs (ASPs) have been developed to limit unnecessary antimicrobial use through reassessment of diagnosis and therapy within 48 to 72 hours, streamlining/de-escalation of antibiotics based on culture/sensitivity data, and the use of antibiotics for the shortest duration needed (Petrak et al. 2003; Dellit et al. 2007). Clinical pharmacists, with the help of infectious disease (ID) team, have an integral role in a day-to-day practice for the judicious use of antibiotics within a short period of time thus promoting further patient safety (Septimus and Owens 2011; ASHP 2010).

Most of inpatients are prescribed intravenous (IV) antibiotics for an extended duration of therapy while an oral (PO) route is possible. Approximately one third of all inpatients initiated on IV antibiotics are eligible for switch to an oral equivalent (McLaughlin et al. 2005). Short intravenous course of therapy for 2–3 days followed by oral medications to complete the course of therapy is beneficial to many patients except in conditions of serious/life-threatening infections, in critically ill patients, or in the presence of contraindications to oral administration as in case of nothing per os (NPO) or comatose patients (Septimus and Owens 2011; ASHP 2010; Bond and Raehl 2005; Bailey et al. 1997; Fischer et al. 2003). Selected oral medication produces serum levels comparable to those achieved through IV form with equivalent clinical outcomes as reduction in the length of hospital stay (LOS) and the duration of IV therapy, along with fewer complications, less patient inconvenience, less healthcare costs and earlier hospital discharge (Septimus and Owens 2011; Lee et al. 2012; Sevinç et al. 1999).

IV to PO conversion guidelines, a component of ASPs, are implemented in hospitals of developed countries and constitute a strategy in daily practice, whereas few hospitals in Lebanon adopt these programs (Septimus and Owens 2011; Kuper 2008). Patients fulfilling the predefined criteria for switch are evaluated. These criteria are based on both drug and host factors (ASHP 2010). Drug factors include high degree of activity against presumed/ known pathogens, high oral bioavailability, low resistance potential, and tolerance with a good safety profile (McLaughlin et al. 2005). Host factors require sufficient absorption of an oral antibiotic with no impairment of gastrointestinal tract and stable clinical condition (Septimus and Owens 2011; Kuper 2008). Improvement of clinical status is evaluated according to temperature, white blood cell count, absence of signs of sepsis, as well as specific criteria for particular infections (Septimus and Owens 2011; Bond and Raehl 2005; Kuper 2008; Gross et al. 2001; Kuti et al. 2002).

There exist three types of IV to PO conversion therapy: sequential, switch and step-down therapies (Kuper 2008). A sequential therapy consists of converting from IV to PO agents with the same compound; a switch therapy is converting with an identical potency; and a step-down therapy is converting with a reduced potency (Kuper 2008). The choice of the type depends on the antimicrobials class and the availability of an oral equivalent (Septimus and Owens 2011).

Most of the studies conducted for the evaluation of IV to PO antimicrobial conversion were interventional studies (Bailey et al. 1997; Kuti et al. 2002; Davis et al. 2005; Ho et al. 2005; Yen et al. 2012; Halley 2000), but have been restricted to certain antibiotic classes (Fischer et al. 2003; Kuti et al. 2002; Davis et al. 2005; Ho et al. 2005; Yen et al. 2012; Pablos et al. 2005; Buyle et al. 2010; Marra et al. 2000) and certain medical conditions (Davis et al. 2005; Cunha 2001; Ramirez and Bordon 2001; Fine et al. 2003). All of which were mostly allocated in the USA (Davis et al. 2005; Galanter et al. 2010; Lau et al. 2011; Glemaud 2000; Fox et al. 2003), others were done in Canada (Ho et al. 2005; Marra et al. 2000; Frighetto et al. 1995; Malfair et al. 1996; Zamin et al. 1997), Ireland (Dunn et al. 2011, Al-Eidan et al. 1999, Chan et al. 1995), Spain (Castro-Guardiola et al. 2001; Martínez et al. 2000), Netherlands (Oosterheert et al. 2006) and Switzerland (Mertz et al. 2009; Senn et al. 2004). These studies demonstrated comparable clinical efficacy rates without serious adverse events and improvements in therapeutic and financial outcomes (Fischer et al. 2003; Ho et al. 2005; Galanter et al. 2010).

The aim of the study was to evaluate the practice of switching from IV to PO antibiotics based on predefined eligibility criteria and its impact on clinical outcomes as the duration of IV antibiotic therapy and LOS, and to assess the correlation between the type of conversion and antibiotic classes.

## Methods

### Study design and study population

This was a retrospective observational study conducted in three Lebanese university teaching hospitals. Patient’s information was obtained from the medical records of each site. All patients hospitalized for more than 24 hours were screened for possible inclusion. The Institutional Review Board of the three hospitals and the School of Pharmacy at the Lebanese International University approved the study.

### Inclusion and exclusion criteria

Adult inpatients receiving an IV antibiotic for more than 48 hours were included. Excluded from the analysis were patients: younger than 18 years of age, not eligible for oral formulation based upon a permanent physiologic condition (e.g. malabsorption syndrome, partial or total removal of stomach, or short bowel syndrome), patients with malignancies or admitted to cardiac/intensive care and surgery units. In addition to those who received IV prophylactic antibiotics, or if a prolonged course of IV antibiotics was required as in case of osteomyelitis, meningitis, Staphylococcus aureus bacteremia and endocarditis.

### Data collection

A structured questionnaire was developed and data were recorded by the principle investigator without any interference on results. This questionnaire has been previously validated by the School of Pharmacy. It is divided into three parts. The first part included demographic characteristics of patients, co-morbidities, allergies, primary diagnosis or presumed indication for antibiotic therapy, microbiological results if available. The second included the antibiotics administered, specifying the type, route of administration, duration of IV therapy, and LOS. Additional data were recorded if a switch from the initial prescribed antibiotic was done, this included the time of switch along with the type of modification whether discontinuation or conversion from IV to PO therapy. In the third part, daily recording of signs and symptoms to assess clinical stability throughout the hospital stay (hemodynamic stability as temperature, blood pressure, heart rate, white blood cell count, respiratory rate, and additional criteria for specific infections as assessment of cough, dyspnea and oxygen saturation for respiratory tract infections, and redness, heat and induration for skin and soft tissue infections).

### Outcomes

The primary outcome of this study was to evaluate the practice of the utilization of IV antibiotic therapy through an assessment of the switch according to predefined criteria for clinical stability. Criteria for IV to PO switch was defined as patients who had received an IV antibiotic for ≥ 48 hours and were able to tolerate oral therapy; no vomiting or diarrhea or NPO; and clinical improvement with temperature less than 38°C, systolic blood pressure >90 mmHg, heart rate <100 beats per minute, and normal white blood cell count or a decrease of at least 2000 cells/μL over the last 24 hours. A transition to PO antibiotics was not expected when a patient’s condition was unstable and in the case of erratic gastrointestinal absorption that can be due to either severe acute diarrhea or vomiting.

Secondary outcomes were to evaluate the duration of IV therapy and LOS, and to identify the type of conversion used in relation to antibiotic classes when a switch was done.

### Statistical analysis

Statistical analysis was performed using the Statistical Package for the Social Science software (SPSS version 20.0). Descriptive statistics were used to describe patient characteristics (frequencies and percentages for categorical variables), and mean (± standard deviation) for continuous variables. The 2 groups of patients (i.e. converted and not converted) were compared for statistically significant differences using chi-square or Fisher’s exact tests for categorical variables, and student-*t*-test for continuous variables (length of IV therapy and LOS), as appropriate. All reported p-values were two-sided with the alpha set at a significance of 0.05.

## Results

### Patient characteristics

**Figure 1:**
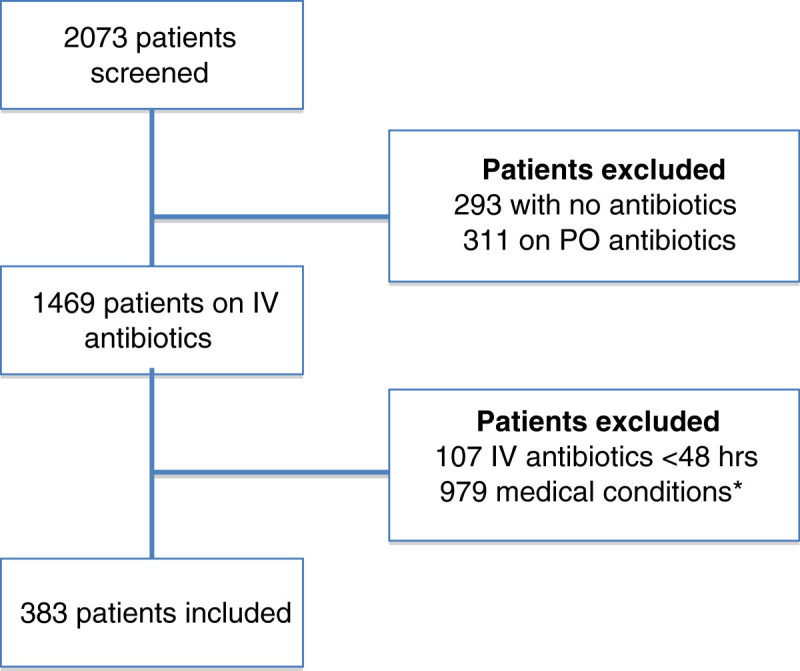
**Flowchart for identification of patients eligible to the study.** The identification of patients eligible for the inclusion criteria in the study was done in 2 steps. *Medical conditions for excluded patients include pediatrics, surgery and care units, malignancies, severe/life-threatening infections, acute respiratory distress syndrome, central nervous system/seizures/meningitis, and gastrointestinal problems. Abbreviations: IV, intravenous; PO, oral.

The study was performed over a period of six months from December 2013 to May 2014 in three Lebanese university teaching hospitals. A total of 2073 patient admissions were screened for inclusion, out of which 604 were excluded. The most common reasons for exclusion were either the patients did not receive IV antibiotics or received PO antibiotics. The remaining 1469 patients taking IV antibiotics were further reviewed for inclusion in the study out of whom 383 patients treated with 491 antibiotic courses met the inclusion criteria as shown in Figure 1. Of these patients, 79 (20.6%) were on concurrent IV and PO antibiotics and 304 (79.4%) were on IV antibiotics only.

The characteristics of study sample are shown in Table 1. Overall the mean age in years (± SD) of the patients was 59.68 (±18.99) years with 198 (51.7%) males. From the enrolled patients, 261 (68.1%) had one or more co-morbidities. The most frequent co-morbidities encountered were hypertension (176, 46%) and diabetes mellitus (144, 37.6%). All included patients were treated on the ward to which they were initially admitted for at least 3 days with IV antibiotics for at least 48 hours. The most frequent site of infection was the respiratory tract infections (188, 49.1%), followed by COPD exacerbation (99, 25.8%).

**Table 1:** **Characteristics of study sample of switch eligible patients**

Characteristic	Frequency (%)
Age (mean ± SD)	59.68 ± 18.99
Sex	
Male	198 (51.7)
Female	185 (48.3)
Smoking	197 (58.3)
Allergy	
Food	4 (8.5)
Drug	15 (31.9)
Asthma	28 (59.6)
Co-morbidities	
Diabetes mellitus	144 (37.6)
Hypertension	176 (46)
CAD	76 (19.8)
COPD	94 (24.5)
Others	118 (30.8)
Presumed or documented diagnosis	
Respiratory tract infections	188 (49.1)
COPD exacerbation	99 (25.8)
Urinary tract infections	58 (15.1)
Skin & soft tissue infections	40 (10.4)
Gastrointestinal infections	24 (6.3)
Other infections	5 (1.3)

*Abbreviations:* *CAD* coronary artery diseases, *COPD* chronic obstructive pulmonary disease, *SD* standard deviation, *%* percentage.

Prescribed antibiotic classes used for empirical therapy were mostly β-lactams (335, 68.2%), followed by fluoroquinolones (80, 16.3%). Patients received empirical treatment either as monotherapy (281, 73.4%), or combination of more than one IV antibiotic (102, 26.6%) with cephalosporins (193, 39.3%) being the most commonly prescribed antibiotics in the combination regimens. Microbiologically documented infections accounted for 18% of the prescriptions.

## Modifications of antibiotic prescriptions

### Switch from IV to PO therapy

Twenty-seven patients on thirty-nine (7.9%) antibiotic courses did not meet the eligibility criteria for the switch from IV to PO route and required to continue IV antibiotics. A total of 356 patients on 452 IV antibiotic courses met the eligibility criteria for switch and were assessed. The IV antibiotic courses were divided into 300 (66.4%) β-lactams, 33 (7.3%) macrolides, 78 (17.3%) fluoroquinolones, 25 (5.5%) metronidazole, and 16 (3.5%) aminoglycosides and glycopeptides. Out of the total 452 antibiotic courses, 334 (73.9%) were not converted and IV antibiotics were administered beyond day 3, and 118 (26.1%) of IV antibiotics were switched to PO route and therefore assumed eligible for conversion and met the criteria for conversion with a p-value <0.0001. The percentage of patients switched in a timely fashion was comparable between the infection types with p-value >0.05 with the lowest for skin and soft tissue infections (SSTI). The majority of switch from IV to PO alternative (111, 94.1%) was done early in therapy within 3 to 5 days of hospital admission and 7 (5.9%) episodes were delayed in the transition beyond day 5 of admission.

### Correlation between antibiotic classes and switch from IV to PO therapy

**Figure 2:**
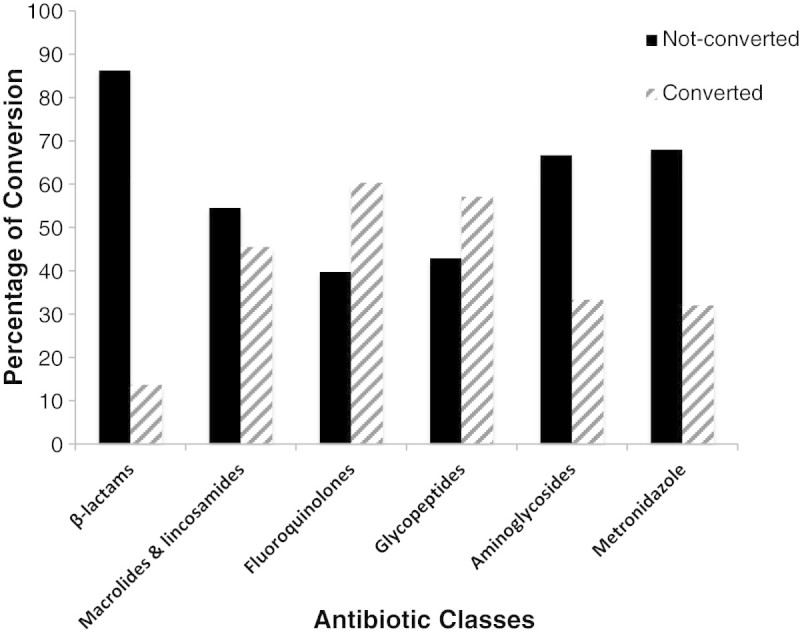
**Frequency of IV to PO conversion among different antibiotic classes in patients eligible for switch.** The percentage of IV to PO conversion was compared among different antibiotic classes in patients eligible for switch in both the converted and non-converted groups. P values were calculated by comparing converted and non-converted antibiotics for each class; P-value <0.05, statistically significant for the conversion of β-lactams (p-value <0.001), macrolides & lincosamides (p-value = 0.009) and fluoroquinolones (p-value <0.0001). Chi-square test used for β-lactams, macrolides and fluoroquinolones, and metronidazole. Fisher’s exact test used for glycopeptides and aminoglycosides. Abbreviations: IV, intravenous; PO, oral.

The associations between antibiotic classes and the frequency of IV to PO switch eligible medication records were studied as shown in Figure 2. Fluoroquinolones were the antibiotic class mostly converted although other classes were eligible for switch. From the patients who were converted to PO agent, conversion was distributed among classes as follows: 13.7% for β-lactams, 45.5% for macrolides, 60.3% for fluoroquinolones, 32% for metronidazole, and 5.9% of aminoglycosides and glycopeptides.

### Correlation between pathogen-specific cultures and switch from IV to PO therapy

During the evaluation period, 99 patients had positive cultures. The correlation between the frequency of switch and microbiologically documented infections was statistically significant where switch over from IV to PO therapy was done more in case of a confirmation of the pathogen responsible for the infection with a p-value of 0.035 as shown in Table 2.

**Table 2:** **Correlation between pathogen-specific cultures and the frequency of switch from IV to PO antibiotics**

	Not switched	Switched
	N(%)	N(%)
Negative culture	306 (82)	86 (72.9)
Positive culture*	67 (18)	32 (27.1)

*P-value = 0.035.

Chi-square test used for statistically significant differences.

*Abbreviations:* *n* frequency, *%* percentage, *IV* intravenous, *PO* oral.

### Type of IV to PO conversion

IV therapy was modified either by discontinuation or switching to oral therapy – in the 118 episodes as shown in Table 3. Out of the 118 episodes, antibiotic treatment was discontinued in 36 antibiotics courses (30.5%) and a switch to oral agents was done in 82 (69.5%) of antibiotic courses based on the predefined clinical criteria. The mean time of switch was 3.81 days (± 1.15) at 95% CI (3.60 – 4.02).

**Table 3:** **Characteristics of IV to PO conversion therapy types in patients that were switched to oral agent and its effect on the length of IV therapy and length of stay**

Type of conversion	Frequency, %
Switch over*	
Sequential	62 (52.5)
Switch	2 (1.7)
Step-down	18 (15.3)
Discontinuation	36 (30.5)
Days of IV therapy (mean ± SD)	2.86 ± 1.16
Length of hospital stay (mean ± SD)	6.56 ± 0.24

Chi-square and Fisher’s exact tests used for statistically significant differences.

Student t- test was used to analyze the length of hospital stay and duration of IV.

*P-value <0.0001.

*Abbreviations:* *IV* intravenous, *PO* oral, *SD* standard deviation, *%* percentage.

**Figure 3:**
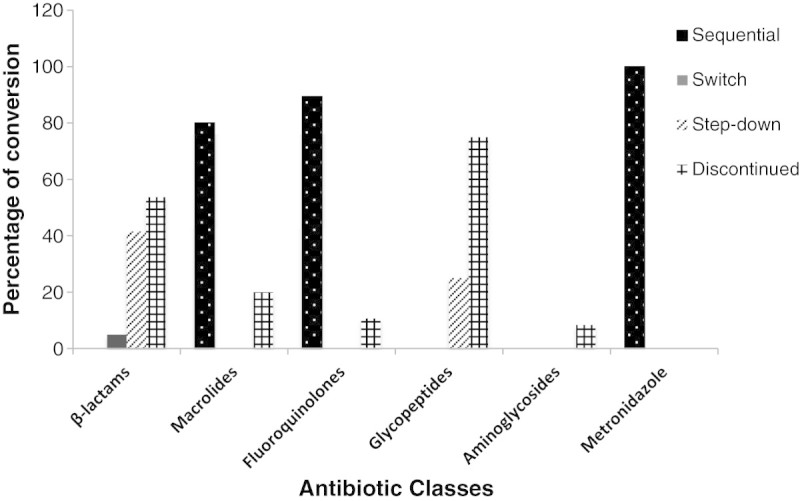
**Percentage of conversion based on the different types of conversion therapy from IV to PO among the antibiotics classes studied.** The percentage of conversion was done based on the different types of conversion therapy from IV to PO (sequential, switch, and step-down) among the different antibiotics classes studied. Statistical significance was observed with β-lactams and fluoroquinolones (p <0.0001), and macrolides (p = 0.002) while no statistical significance (p > 0.05) obtained for the other classes. Fisher’s exact test was used for analysis. Abbreviations: IV, intravenous; PO, oral.

The most frequent type of conversion used in this study was the sequential conversion therapy and the most antibiotic drug classes involved were fluoroquinolones (87.5%), macrolides (80%) and metronidazole (100%) with a p-value of <0.0001, for which both IV and PO formulations are available for the same drug as illustrated in Figure 3.

### Duration of IV therapy and length of hospital stay

**Figure 4:**
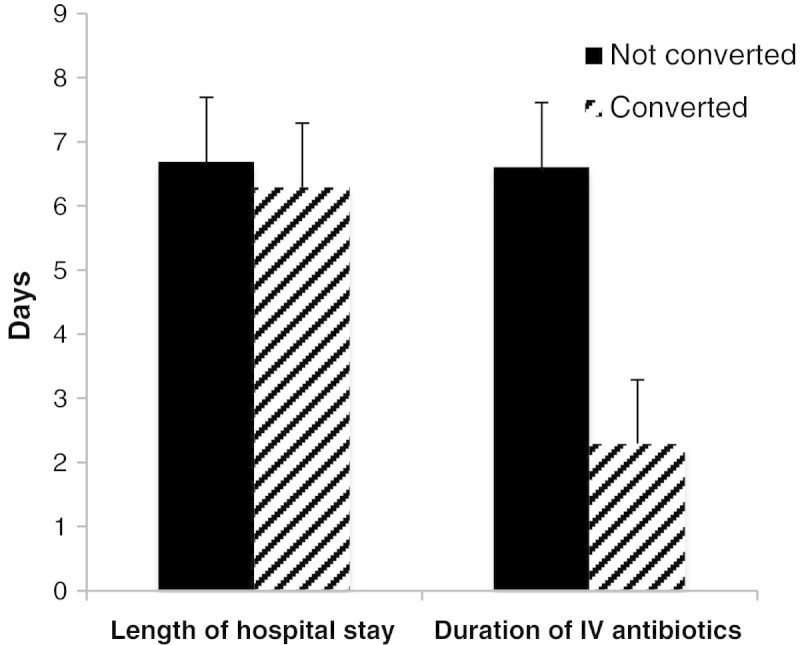
**Duration of IV therapy & length of hospital stay for converted and not converted medication records.** Comparison between the duration of IV therapy and length of hospital stay for converted and not converted medication records. Statistical significance was observed with a P < 0.0001 for the duration of IV therapy and no statistical significance with a p-value = 0.227 for the length of hospital stay. Student t- test was used to analyze the length of hospital stay and duration of IV antibiotics. Abbreviations: IV, intravenous.

Overall, IV antibiotic therapy was continued for 5.39 ± 2.63 days and the LOS in days was 6.63 ± 2.74. Comparing the 2 groups – converted versus not-converted medications records, the LOS was unchanged with no statistical significance (p-value of 0.227); 6.61 days for the 118 patients treated with early switch versus 6.69 days for those who remained on IV treatment. Nevertheless, there was a trend towards a shorter overall duration of antibiotic treatment as shown in Figure 4.

As expected, the duration of IV therapy of converted medication records was shorter than those that were not converted with p-value <0.0001 (95% CI, 2.73 – 3.74).

### Combined use of IV antibiotics and PO medications

Because the ability to tolerate oral medications was used as one of the criteria for switching patients, it was worthwhile to record the number of PO antibiotic prescriptions received by patients on IV therapy and was 79 (20.6%). Furthermore, the fact that patients received oral drugs while on IV antibiotic therapy indicated that these patients had no gastrointestinal absorption problems, however only 12 (10.2%) of those patients were switched with no statistical significance with a p-value >0.05.

## Discussion

This study was done on the Lebanese population where there is a need for unified local guidelines to the physicians and clinical pharmacists to aid in the conversion from IV to PO route when the condition of the patient has improved after few days of hospital admission. Various studies covered IV to PO conversion and their implications in clinical practice as the reduction of the prolonged use of IV antimicrobials and shortening of the LOS. The present study was not designed to prove that an early switch to PO therapy has the same efficacy as a full IV course.

Most of the admitted patients, who are initially prescribed IV antibiotics, can be switched to an appropriate PO formulation once the clinical stability markers are met, provided that the total course of therapy duration is completed. Using the defined criteria for IV to PO switch, two-third of the included patients in the study were not switched to PO therapy, despite improvements in clinical signs of infection. The number of patients that were switched to PO therapy could have been underestimated since the study design did not follow up discharged patients. In the Lebanese practice, patients are converted to PO antibiotic therapy once discharged home, although a 24 hour-period after switch is required for patient’s monitoring before discharge. Other possible barriers to a timely switch strategy perceived by the study results include unfamiliarity with guideline recommendations, misconceptions or lack of outcome expectancy. These barriers were also reflected by the study results of Schouten et al. (Schouten et al. 2007) and Halm et al. (Halm et al. 2001). Physicians were not aware of the existence of clear guidelines on the adequate timing of the switch. Indeed, participating hospitals did not provide these guidelines through booklets or educational sessions and did not adopt ASPs that may be applied in few centers in Lebanon.

Modifications in IV antibiotics were done in one-third of the antibiotic courses; interventions for switch were either through discontinuation of therapy or conversion to the alternative oral drug depending on the antibiotic class that was prescribed. Administered antibiotic courses of therapy that were switched to a suitable PO formulation were few and involved mostly fluoroquinolnes, macrolides and metronidazole classes probably because these antibiotics are available in both PO and IV formulations where sequential conversion type is used. Whereas the number of antibiotic courses on β-lactams (specifically third-generation cephalosporins) switched to a PO alternative was rare, and modification was done through discontinuation of the drug on the day of clinical stability rather than switching to PO therapy, similarly to the observation done by Hunter and Dormaier (Hunter and Dormaier 1995). As a further illustration about the switch, drugs as ceftriaxone with no definitive oral equivalent; its conversion to a PO agent is done through step-down conversion therapy, which was minimally done in this study.

The mean number of days to clinical stability and therefore the time for switch in this study was 3.81 days, correlated with the results of previous studies (Mertz et al. 2009; Senn et al. 2004; Athanassa et al. 2008) that also reported the appropriate time for IV therapy to be reassessed between 2.0 - 4.0 days. In this study, the mean length of IV therapy was around 7 days for non-converted patients compared to around 4 days for the converted ones. However, a reduction in the LOS was not observed, similarly to Laing et al. (Laing et al. 1998) even though the duration of IV therapy shortened in patients who were converted which correlates with the results of previous studies (Mertz et al. 2009; Laing et al. 1998).

Antibiotic resistance is directly related to the excessive use of antibiotics. Previous studies evaluating antibiotic use in hospitals have shown that up to 50% of prescriptions can be inappropriate (Goldmann et al. 1996; McGowan 1994; Kollef et al. 1997; Thuong et al. 2000). Hence limiting the misuse of antibiotics may be an important intervention for reducing levels of resistance. Results of the study confirm the tendency of physicians to prescribe broad-spectrum antibiotic therapy empirically with β-lactams mainly third-generation cephalosporins followed by fluoroquinolones the most widely used antibiotic classes among assessed patients. Therefore, improving appropriateness of therapy at the time of initial empirical prescribing is a difficult challenge associated with many organizational requirements and governed by the hospital policy.

### Strengths and limitations

The strengths of the study were that it was a multicenter study where a large number of patients were screened. The principle investigator recorded data without any interference or bias. The study also assessed all classes of antibiotics and different types of infections and highlighted the vital role of clinical pharmacist to hospital practice. Clinical pharmacists can follow a defined protocol to daily assess patient eligibility for an IV to PO switch, be in charge of the appropriate route of the medication, and monitor the patient’s progress and tolerability.

This study has several limitations. First, the study was observational while an interventional study could have a greater impact on the results. Second, lack of unified documentation of patients’ information among the three medical sites (paper based or computerized databases), as well as difference in the physicians’ educational background. However the lack of uniformity between the sites and the teams demonstrated that the evaluation tool could be effectively used in a variety of clinical settings. Third, although criteria for clinical stability were defined and previously set to assist in the evaluation of appropriate IV to PO conversion, the impact of other factors that influenced clinical decisions (e.g. lack or missing data for daily eligibility criteria, reasons for non-conversion, complications due to co-morbidities) were not assessed.

## Conclusion

The results of this evaluation highlighted the need of a structured approach and clear guidelines to appropriately prescribe antibiotics and review the therapeutic decisions including assessment for improvement in patient’s clinical status for antibiotic conversion from IV to PO therapy in daily practice. Further studies are needed to assess physician’s knowledge, beliefs, and acceptance of the switch over from IV to PO therapy in order to establish an effective and unified guideline for IV to PO conversion and consequently its implementation through team approach. This study did not measure the reduction in complications from the IV to PO switch since it was a retrospective observational study. Hence, this raises the need for a future prospective research with a pharmacist-involved intervention.

## Authors’ original submitted files for images

Below are the links to the authors’ original submitted files for images. Authors’ original file for figure 1 Authors’ original file for figure 2 Authors’ original file for figure 3 Authors’ original file for figure 4
