# Supplementary material for: Practice of switch from intravenous to oral antibiotics
Source: Springerplus. 2014 Dec 9;3:717. doi: 10.1186/2193-1801-3-717 (PMC4320166; doi:10.1186/2193-1801-3-717)
Supplement: Supplementary file 1 — Authors’ original file for figure 1 [file 40064_2014_1490_MOESM1_ESM.pdf]

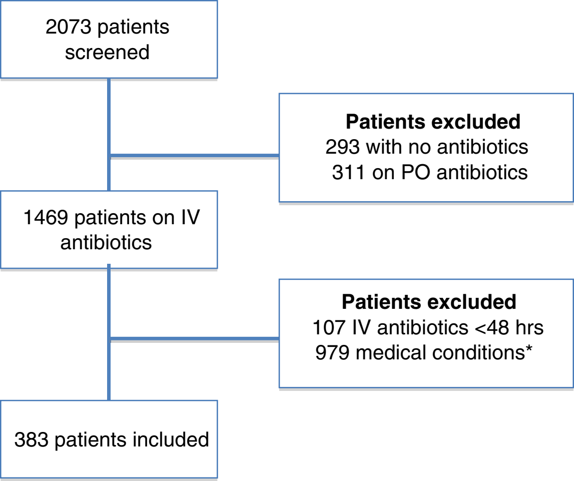
2073 patients screened
Patients excluded
293 with no antibiotics
311 on PO antibiotics
1469 patients on IV antibiotics
Patients excluded
107 IV antibiotics <48 hrs
979 medical conditions*
383 patients included
