# Supplementary figures and images for: Practice of switch from intravenous to oral antibiotics
Source: Springerplus. 2014 Dec 9;3:717. doi: 10.1186/2193-1801-3-717 (PMC4320166; doi:10.1186/2193-1801-3-717)

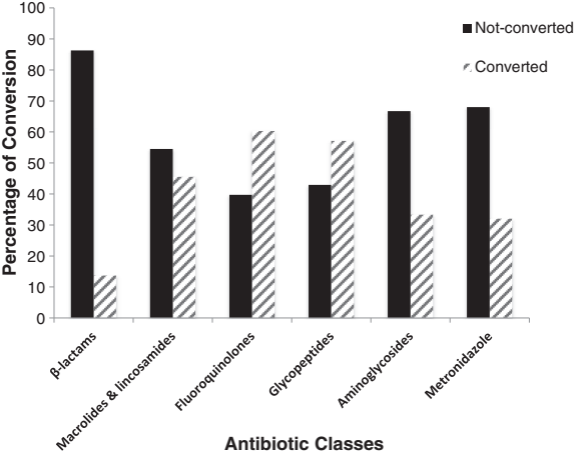

Supplement: Supplementary file 2 — Authors’ original file for figure 2 [file 40064_2014_1490_MOESM2_ESM.pdf]

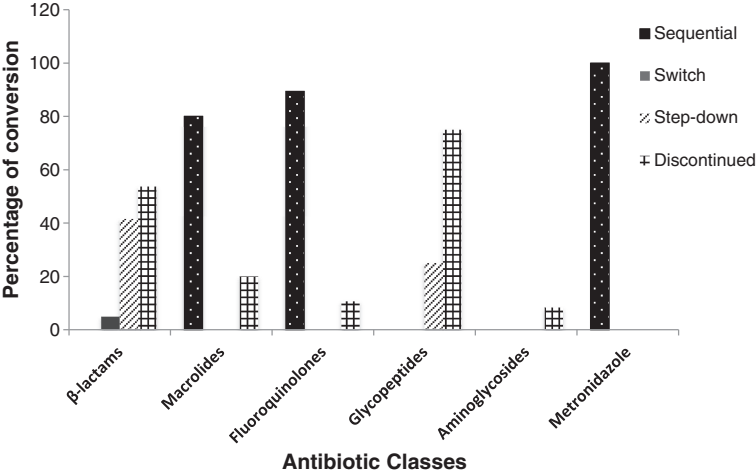

Supplement: Supplementary file 3 — Authors’ original file for figure 3 [file 40064_2014_1490_MOESM3_ESM.pdf]

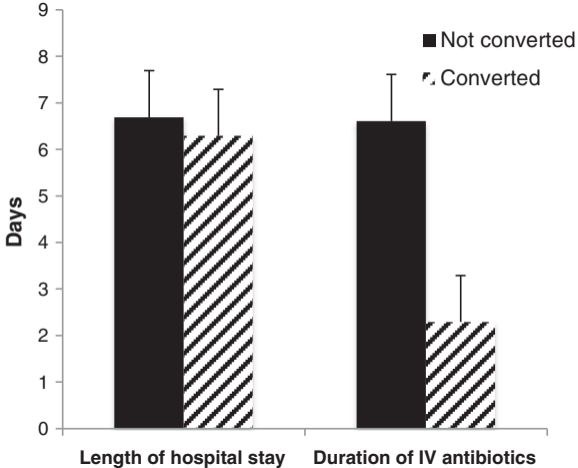

Supplement: Supplementary file 4 — Authors’ original file for figure 4 [file 40064_2014_1490_MOESM4_ESM.pdf]
